# Control of immune cell entry through the tumour vasculature: a missing link in optimising melanoma immunotherapy?

**DOI:** 10.1038/cti.2017.7

**Published:** 2017-03-17

**Authors:** Lih Yin Tan, Carmela Martini, Zvi G Fridlender, Claudine S Bonder, Michael P Brown, Lisa M Ebert

**Affiliations:** 1 Centre for Cancer Biology, University of South Australia and SA Pathology, Adelaide, SA, Australia; 2 School of Pharmacy and Medical Sciences, University of South Australia, Adelaide, SA, Australia; 3 Institute of Pulmonary Medicine, Hadassah-Hebrew University Medical Center, Jerusalem, Israel; 4 Cancer Clinical Trials Unit, Royal Adelaide Hospital, Adelaide, SA, Australia; 5 Discipline of Medicine, University of Adelaide, Adelaide, SA, Australia

## Abstract

Metastatic melanoma remains a fatal disease to many worldwide, even after the breakthrough introduction of targeted therapies such as BRAF inhibitors and immune checkpoint blockade therapies such as CTLA-4 and PD-1 inhibitors. With advances in our understanding of this disease, as well as the increasing data gathered from patient studies, the significance of the host immune response to cancer progression and response to treatment is becoming clear. More specifically, the presence of intratumoral CD8^+^ cytotoxic T-cells correlates with better prognosis whereas the accumulation of monocytes/macrophages and neutrophils in the tumour is often associated with worse prognosis. Access and infiltration of circulating leukocytes into the tumour is governed by adhesion molecules and chemokines expressed by the endothelial cells of the vasculature. This review focuses on the adhesion molecules and chemokines which control the homing of CD8^+^ cytotoxic T-cells, monocytes and neutrophils to peripheral tissues, including tumours. We discuss the role of these leukocyte subsets in regulating melanoma growth, and detail the mechanisms used by tumours to selectively recruit or exclude these leukocytes for their own advantage. In doing so, we bring to light an underappreciated component of tumour biology which should be considered in combination with current treatments to selectively alter the leukocyte composition of tumours and ultimately enhance treatment outcome.

## Melanoma: a paradigm for understanding immune control of cancer

Melanoma is the deadliest form of skin cancer, contributing to 75% of skin cancer related fatalities. Its high somatic mutation rate and consequent formation of multiple neoantigens are thought to be responsible for the exceptional immunogenicity of this malignancy.^1^ Hence melanoma is the best studied cancer in terms of its interaction with the immune system and response to immunotherapy, and will therefore be the focus of this review. In particular, there is increasing evidence that the tumour endothelium, which controls the entry of leukocytes from the bloodstream into the tumour, acts as a therapeutic barrier. Here, we review the known endothelial adhesion molecules and chemotactic factors involved in differential recruitment of CD8^+^ T-cells, monocytes and neutrophils into melanoma tumours, and present clinical evidence that these molecules and factors are crucial in affecting immunotherapy response and outcome of melanoma patients. Finally, we suggest combination therapies based on promising preclinical data to modify the expression of these molecules and factors to enhance immunotherapy response in melanoma patients.

## The metastatic potential of cutaneous malignant melanoma

The approaches used to treat melanoma, and the efficacy of such treatments, are highly dependent on the stage of disease progression. If detected during the radial growth phase while the tumour is still confined to the upper epidermal layers of the skin, a simple surgical excision is generally sufficient to fully cure the disease. However, once the melanoma progresses into the vertical growth phase, invades the deeper dermal layers of the skin, and gains the potential to metastasise to draining lymph nodes or other organs via the bloodstream, surgery is less likely to be a curative treatment modality. Radiotherapy has a limited role in the adjuvant setting and may provide useful palliative treatment for metastatic lesions. Over the last 5 years or so, new targeted therapies using small-molecule kinase inhibitors and immune checkpoint inhibitory antibodies have largely supplanted cytotoxic chemotherapy. These kinase inhibitors include vemurafenib and dabrafenib (BRAF inhibitors; BRAFi) and trametinib (MEK inhibitor), which attenuate the signalling flux of the mitogen-activated protein kinase pathway that is constitutively active in ~90% of melanoma.^2^ Nevertheless, despite initially rapid and deep tumour responses in most metastatic melanoma patients, these agents have the major drawback of drug resistance, which limits the median progression-free interval to approximately 9–10 months.^2^

## Immunotherapy for unresectable metastatic melanoma

The targeted mitogen-activated protein kinase inhibitors benefit the minority (up to 40%) of metastatic melanoma patients whose diseases carry an oncogenic BRAF mutation. On the other hand, the breakthrough development of checkpoint blockade immunotherapy (also within the last ~5 years) is expected to have a major impact in improving long-term patient survival. Current Food and Drug Administration approved immunotherapies for metastatic melanoma include antibody inhibitors of the CTLA-4 checkpoint molecule (ipilimumab) or the PD-1 checkpoint molecule (nivolumab and pembrolizumab), although many others are currently in clinical development. CTLA-4 blockade promotes the priming and activation of tumour-specific CD8^+^ T-cells, whereas PD-1 blockade reinvigorates the cytotoxic function of intratumoral CD8^+^ T-cells.^3^ Currently, the tumour response rates for these inhibitors when used as monotherapy are ~20 and ~40%, respectively. Now, emerging data from ongoing clinical trials indicates even higher response rates of ~60% from the combinations of ipilimumab and nivolumab^4^ or ipilimumab and pembrolizumab.^5^ Strikingly, many patients who respond to this form of immunotherapy exhibit deep and durable remissions of disease, which has changed a diagnosis of metastatic melanoma from a virtual death sentence to the prospect of cure. However, we currently have a very limited understanding of the factors which control responsiveness to checkpoint blockade therapy, and improving response rates will be a critical focus over the coming years.

An experimental kind of immunotherapy called adoptive cell transfer (ACT) reviewed in ref. 6 may also have role in the treatment of metastatic melanoma especially where other approaches have failed or for melanoma subtypes such as uveal melanoma, which seem to be less responsive to immune checkpoint blockade. ACT involves the reinfusion of autologous and *ex vivo* expanded tumour-specific CD8^+^ T-cells into the body following lymphodepleting chemotherapy or radiotherapy. In non-intent to treat analyses, tumour response rates of up to 72%, with 20% of the patients experiencing durable complete regressions, have been reported.^7^ Patients responding to ACT were responsive regardless of previous treatments,^7^ highlighting the value of continuing to investigate and improve this treatment approach. Alternatively, the autologous CD8^+^ T-cells can be genetically engineered to express either tumour antigen-specific T-cell receptors (via TCR gene transfer) or chimeric antigen receptors (CAR T-cells) before ACT.^6^ Although CAR T-cells have demonstrated striking success in CD19-expressing haematological malignancies with objective response rates of ~90%,^6^ the effectiveness of this approach in melanoma and other solid tumours remains under investigation. For melanoma in particular, GD2-specific CAR T-cells are currently in phase 1 clinical trial for metastatic melanoma patients.^8^

## The nature of the immune cell composition in tumours is linked to melanoma prognosis

Although the aforementioned approaches have all shown promising results, not all patients respond to immunotherapy. Recently, Blank *et al.*^9^ introduced the concept of an immunogram, highlighting the seven key factors underlying immunotherapy response. Not surprisingly, immune cell infiltration is one of them. Indeed, although peripheral blood levels of immune cells can be a useful biomarker of disease progression, the immune cells need to be physically in the tumour to exert their functions. Accordingly, the degree of infiltration of lymphocytes (tumour-infiltrating lymphocytes; TIL), and in particular activated CD8^+^ T-cells, within melanoma positively correlates with better prognosis.^10, 11, 12^ Of note, CD8^+^ T-cell activity following infiltration into the tumour may be inhibited. This may explain why the detection of CD8^+^ T-cells within patient samples in the absence of information about activation marker expression has led to discrepancies in its correlation with patient outcome. Another point to note is, apart from CD8^+^ T-cells, the infiltrating lymphocytes in melanoma can also include CD4^+^ T-cells, a variable proportion of which are regulatory T-cells and which have been found to be negatively associated with melanoma progression.^13^ Their recruitment and role in immunotherapy has been reviewed elsewhere^14^ and will not be covered in this review.

As highlighted in the immunogram, the presence of other immune cell types has also been found to correlate with disease progression and response to therapy. This is due to their contribution in both influencing CD8^+^ T-cell function and further shaping the local tumour microenvironment to either a pro- or anti-tumorigenic condition. One example is tumour-associated macrophages (TAM). TAM can be polarised upon IFNγ stimulation into a M1 phenotype, which exhibit enhanced anti-tumorigenic properties; or into a M2 phenotype upon IL-4 stimulation, which exhibit pro-tumorigenic activities.^15^ Immunohistochemistry on primary melanoma biopsies revealed an increase in TAM infiltration with melanoma progression,^16^ which also correlated with increased melanoma invasiveness and metastasis.^17^ Interestingly, from a small cohort of metastatic melanoma patients treated with BRAFi, higher numbers of TAM before treatment correlated with shortened progression-free survival.^18^

In addition to TAM, the importance of tumour-associated neutrophils (TAN) in prognosis and treatment response is also beginning to emerge. We have shown that neutrophils can also be polarised into an anti-tumorigenic N1 phenotype following IFNβ stimulation or into a pro-tumorigenic N2 phenotype following transforming growth factor beta stimulation.^19^ There is also increasing evidence in both human and murine studies that neutrophils inside the tumour are predominantly pro-tumorigenic, especially in advanced tumours.^20, 21^ While our current understanding of TANs and their potential role in tumour progression is in its infancy when compared to CD8^+^ T-cells and TAM, it is likely to develop in the very near future. In a murine melanoma model, it was observed that TAN were more cytotoxic towards primary but not metastatic tumours.^21^ This was further explained in murine Lewis lung carcinoma and mesothelioma models, where TAN gradually shifted from an N1 phenotype to an N2 phenotype with disease progression.^20^ Immunohistochemistry on primary melanoma from patients revealed that the presence of intratumoral neutrophils significantly correlated with poor survival.^22^ Similarly, increased neutrophils in peripheral blood also correlated with poor survival,^23^ non-responsiveness to ipilimumab treatment^24^ and reduced overall survival following ipilimumab treatment^25^ in metastatic melanoma patients.

Both TAM and TAN are closely related to myeloid-derived suppressor cells (MDSC) which are a heterogenous population of immature myeloid progenitors that can be further subdivided into monocytic-MDSC (M-MDSC) and granulocytic MDSC (G-MDSC). Increased M-MDSC and G-MDSC frequencies were observed in advanced melanoma patients,^26^ with lower M-MDSC baseline levels correlating with patient responsiveness to ipilimumab.^27^ It has been suggested that MDSC could be mobilised and recruited to tumours with similar pathways to that of TAM and TAN,^28^ highlighting the importance of TAM and TAN recruitment which are a focus in this review.

Taken together, these findings demonstrate the significance of CD8^+^ TIL, TAM and TAN in dictating melanoma disease progression and treatment outcome, and in turn highlight the importance of controlling the entry of subsets of these immune cells into the tumour, which leads us to consider the role of the endothelium.

## The role of endothelial adhesion molecules in regulating CD8^+^ TIL, TAM and TAN recruitment to melanoma

The endothelium acts as a barrier between the leukocytes in the bloodstream and the tumour microenvironment. In order for leukocytes to infiltrate into the tumour, they have to roll along the endothelium (via the engagement of selectins and their ligands), firmly adhere to the endothelial cells (ECs; through integrin interactions) and subsequently transmigrate across the endothelium into the underlying tissue to elicit their functions.^29^ Leukocytes can only undergo this series of events if they express an appropriate combination of adhesion molecules which are cognate to those expressed by ECs. Accordingly, adhesion molecule expression is tightly regulated on both leukocytes and ECs, and provides the endothelium with a mechanism to selectively recruit leukocyte subsets appropriate for the immune response required (Table 1).

**Figure 1:**
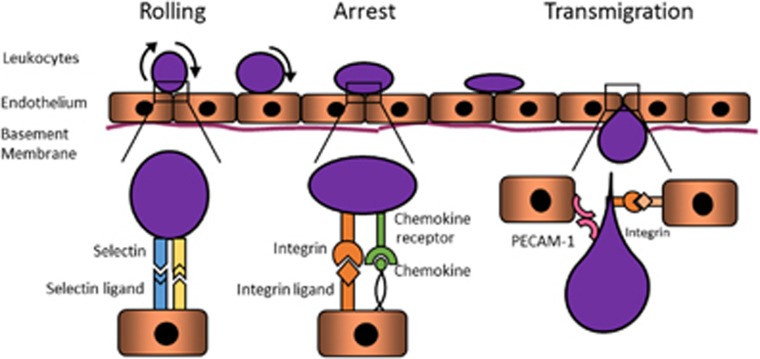
Leukocyte recruitment on the normal endothelium, and the adhesion molecules/ligands which are relevant for each step.

The key endothelial adhesion molecules that regulate leukocyte recruitment include the selectins (P-selectin, E-selectin) for leukocyte rolling, integrin ligands such as ICAM-1, ICAM-2, VCAM-1 and MAdCAM-1 for leukocyte firm adhesion, as well as junctional adhesion molecules and platelet/endothelial cell adhesion molecule-1 (PECAM-1) for leukocyte transmigration^29^ (Figure 1). While the expression levels of these adhesion molecules can be constantly regulated based on environmental stimuli on normal endothelium, tumour ECs are found to be non-responsive (‘anergic') to the pro-inflammatory signals that would normally upregulate adhesion molecule expression. As a result, the endothelium within a melanoma has a distinctly different adhesion molecule expression profile compared to the endothelium of normal, healthy tissue.

Immunohistochemical studies of samples from melanoma patients have revealed that primary melanoma intratumoral vessels have reduced P-selectin expression^30^ and VCAM-1 expression^31^ when compared to non-tumoral blood vessels in adjacent tissues. Furthermore, intratumoral vessels of metastatic melanoma patients also exhibit decreased P-selectin, E-selectin, ICAM-1 and VCAM-1 expression, although these are expressed strongly on non-tumoral blood vessels in adjacent tissue.^30, 31, 32^ This is also consistently observed in B16 murine melanoma models. Lineage tracing performed on the endothelium of tamoxifen-pulsed VE-cadherin-CreERT2 Rainbow mice engrafted with a B16 melanoma revealed the downregulation of gene expression levels of selectin, ICAM-1, VCAM-1 and MAdCAM-1 in melanoma endothelium compared to normal endothelium with disease progression.^33^

Tumour ECs are rendered anergic following chronic stimulation with multiple factors. These include the angiogenic factors vascular endothelial growth factor (VEGF) and basic fibroblast growth factor, transforming growth factor beta, adenosine, as well as nitric oxide. *In vitro*, prolonged VEGF and basic fibroblast growth factor exposure modified ECs epigenetically,^34^ resulting in suppressed adhesion molecule expression, even following pro-inflammatory cytokine tumour necrosis factor alpha stimulation.^35^ Similarly, *in vitro* treatment of IL-1α- or tumour necrosis factor alpha-activated ECs with transforming growth factor beta, adenosine or nitric oxide, respectively, was found to differentially downregulate P-selectin, E-selectin, ICAM-1 and VCAM-1 expression.^36, 37^ Some of these factors are often reported to be upregulated in melanoma with increased disease progression,^38, 39^ thus providing a potential mechanism to explain the low level of adhesion molecule expression observed on ECs in melanoma. Confirmation of the precise mechanism(s), however, awaits further studies.

Given the dependence of T-cell infiltration on these adhesion molecules, downregulation of these molecules would be expected to result in a decreased ability of T-cells to enter tumours. Indeed, in tumours of metastatic melanoma patients, CD8^+^ T-cells preferentially localised at the tumour periphery where there was high P-selectin, E-selectin and ICAM-1 expression and not at areas within the tumour lacking expression.^32^ This was also demonstrated in a murine melanoma model, where increased expression of adhesion molecules was associated with increased T-cell infiltration and tumour regression, and relapsed tumours exhibited loss of adhesion molecule expression which correlated with decreased T-cell infiltration.^40^

In addition, the melanoma endothelium is also found to express molecules that actively block T-cell infiltration. In a B16 murine model, melanoma vessels expressed CD73, the enzyme that produces adenosine;^36^ as mentioned above, adenosine can contribute to the downregulation of EC adhesion molecule expression. Both melanoma cells and ECs also express endothelin B receptor (ETBR) and its ligand endothelin-1,^41^ which acts through nitric oxide to inhibit T-cell adhesion onto the endothelium.^42^ Apart from that, the tumour endothelium also specifically expresses FasL that can directly kill activated CD8^+^ T-cells, thereby resulting in decreased CD8^+^ T-cell infiltration in multiple solid tumours.^43^

Interestingly, while monocytes and neutrophils are also typically recruited via adhesion molecules such as P-selectin, E-selectin, ICAM-1 and VCAM-1, the downregulation of these endothelial molecules in tumours does not appear to correlate with decreased monocyte or neutrophil infiltration. In fact, macrophages were found to constitute the main leukocyte population in melanoma.^44^ This suggests that there are additional adhesion molecules involved in this process. However, there is relatively little known about the specific adhesion molecules involved in selectively recruiting monocytes and neutrophils into tumours. A potential candidate is the peripheral node addressin (PNAd), an L-selectin ligand normally only expressed in secondary lymphoid tissues but found to be aberrantly expressed on the endothelium in 11 of 18 melanoma primary tumours,^45^ which can interact with monocytes and neutrophils expressing L-selectin. Monocytes and neutrophils also express the integrins CD11c/CD18 (p150/95) and CD11d/CD18 which are not expressed on CD8^+^ T-cells and can interact with multiple extracellular matrix proteins.^46, 47^ Alternatively, the tumour endothelium can also express galectin-3, a member of the lectin family that binds specifically to β-galactosides through its carbohydrate-recognition-binding domain. Galectin-3 which has been found to mediate neutrophil recruitment to murine lung in response to infection,^48^ although its ligand on neutrophils is yet to be identified. A galectin-3 ligand, the neuronal adhesion molecule L1 is also expressed on monocytes,^49^ although its potential role in monocyte recruitment is yet to be demonstrated. Interestingly, galectin-3 expression has been found to correlate with T-cell apoptosis in human melanoma biopsies, thereby potentially further inhibiting T-cell recruitment.^50^ Furthermore, galectin-3 has also been found to inhibit T-cell function, in particular IFN-γ secretion, through the inhibition of TCR and CD8 receptor association.^51^ Nonetheless, there is a lack of convincing evidence that these molecules are directly involved in monocyte and neutrophil recruitment in melanoma, thereby highlighting the need for further investigation.

**Figure 2:**
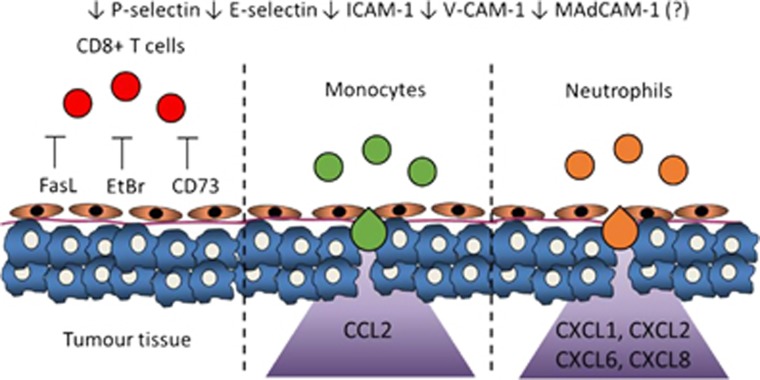
Differential recruitment of CD8^+^ T-cells, monocytes and neutrophils by the tumour endothelium, and associated molecules.

Taken together, the current evidence indicates that there is a downregulation of endothelial adhesion molecules is necessary for recruiting CD8^+^ T-cells on the melanoma endothelium, therefore significantly inhibiting T-cell recruitment (Figure 2).

## The chemokines important for recruitment of CD8^+^ TIL, TAM and TAN to melanoma

In addition to endothelial expressed adhesion molecules, immune cell recruitment also requires a chemoattractant. Chemokines, which are anchored in the glycocalix of the endothelium and are displayed to rolling leukocytes, trigger integrin-mediated firm adhesion, while chemokine gradients generated in the underlying tissue guide the transmigrated leukocytes to the site in need of leukocyte function. Owing to differential expression of chemokine receptors, CD8^+^ T-cells, monocytes and neutrophils can respond to different sets of chemokines.

Of the many chemokines that CD8^+^ T-cells can respond to, those that signal through the CCR5 and CXCR3 axes are suggested to be the most important in recruiting CD8^+^ T-cells into melanoma. These include the CCR5 ligands, CCL3, CCL4 and CCL5, and the CXCR3 ligands, CXCL9, CXCL10 and CXCL11.^52, 53^ Although CCR5 expression is crucial for both the recruitment and effector functions of CD8^+^ T-cells in solid tumours,^53^ there is also a non-redundant role for CXCR3 in controlling CD8^+^ T-cell trafficking to human and murine melanoma, not achievable by CCR5.^52^ This could potentially explain why the overexpression of CCL5 in many tumour models is more often associated with pro- and not anti-tumorigenic outcomes, because of its ability to also promote monocyte infiltration.^54^

Therefore, a critical increase in the signalling ligands for CXCR3 combined with ligands of CCR5 would be expected to induce robust CD8^+^ T-cell infiltration. Indeed, CCL5 and CXCL9 co-expression synergistically led to CD8^+^ T-cell infiltration in a murine melanoma model.^55^ Unfortunately, the CXCR3-signalling ligands are often downregulated with disease progression in melanoma. For example, lineage tracing performed on the endothelium of tamoxifen-pulsed VE-cadheren-CreERT2 Rainbow mice engrafted with a B16 melanoma revealed a downregulation of CXCL9 and CXCL10 gene expression in melanoma endothelium with increased tumour progression.^33^ Furthermore, CXCL10 expression was found to be reduced with metastasis in a lung metastatic melanoma model, in which CXCL9 and CXCL10 expression were both inhibited by adenosine.^56^

More importantly, these *in vivo* observations in mice translate into clinical evidence with expression of CXCL9 and CXCL10 in primary human melanoma correlating with increased CD8^+^ T-cell infiltration.^57^ Similarly, in metastatic melanoma, increased CD8^+^ T-cell infiltration was associated with enhanced expression of CCL3, CCL4, CCL5, CXCL9 and CXCL10.^58^ Furthermore, overexpression of CCR5 and CXCR3 ligands correlated with responsiveness to treatment. More specifically, (i) increased expression of CCL4, CCL5, CXCL9, CXCL10 and CXCL11 correlated with increased likelihood to respond to ipilimumab;^59^ (ii) pre-treatment overexpression of CCL5, CXCL9, CXCL10 and CXCL11 in melanoma patients predicted responsiveness to adoptive cell therapy and interleukin-2;^60^ and (iii) enhanced CCL5, CXCL9 and CXCL10 in tumours following chemotherapy correlated with increased survival and tumour control in melanoma patients.^55^ In accordance with that, CXCR3 expression also correlated with disease progression, where (i) expression of CXCR3 on CD8^+^ T-cells correlated with melanoma patient survival with stage III disease;^61^ and (ii) a retrospective analysis of melanoma patients revealed loss of CXCR3 associated with metastasis.^62^

Although there is an abundance of clinical evidence supporting the importance of the aforementioned chemokines in CD8^+^ T-cell recruitment to melanoma, there are few data from human studies documenting the chemokines important for TAM and TAN recruitment to melanoma. Our current understanding of the candidate chemokines involved in this process is therefore largely based on evidence from animal models.

In mice, a key chemokine for mediating monocyte recruitment to melanoma is CCL2, which signals through the receptor CCR2. CCL2 overexpression in primary murine melanoma led to monocyte infiltration,^63^ and CCL2 blockade in a human melanoma xenograft model led to a reduction in macrophage infiltration.^64^ Although CD8^+^ T-cells also express the receptors for CCL2, post-translational modification of CCL2 such as nitration by reactive nitrogen species in the tumour is found to inhibit CD8^+^ T-cell recruitment via this axis.^65^

The key chemokines involved in TAN recruitment to murine melanoma signal via the CXCR1 and CXCR2 axes, including CXCL1, CXCL2, CXCL6 and CXCL8. B16-F10 murine melanoma was found to express high levels of CXCL1 and CXCL2, therefore leading to recruitment of TAN to the tumour.^66^ Human melanoma xenografts overexpressing murine CXCL6 in mice exhibited increased TAN influx and enhanced tumour growth.^67^ Similarly, CXCL8 overexpression in a human melanoma xenograft model led to increased neutrophil recruitment.^21^ Interestingly, CXCL8 expression is tightly associated with melanoma progression in patients,^68^ thereby prompting speculation that this pathway may contribute to increased N2 neutrophil prevalence with increased disease severity.

Clearly, via expression of other chemokine receptors, TAM and TAN can also be responsive to other chemotactic factors. Indeed, TAM and TAN have been described to be recruited by CCL5, VEGF and CXCL5 in other cancer models.^54, 69, 70^ However, these remain to be elucidated in melanoma. Taken together, the melanoma tumour microenvironment appears to be depleted of chemokines that recruit CD8^+^ T-cells and yet enriched with chemokines that recruit monocytes and neutrophils (Figure 2,Table 2). This is likely a major factor in the creation of a tumour microenvironment that supports tumour growth and inhibits anti-tumour immunity.

## The role of platelets in monocyte and neutrophil recruitment

Platelets are small cellular fragments that are derived from megakaryocytes. Although their primary role is to regulate bleeding and clotting processes under normal conditions, platelets have also been implicated in malignancy. In patients with metastatic melanoma, univariate analysis revealed that low pre-treatment levels of platelets significantly correlated with increased median patient survival time.^71^ Interestingly, emerging evidence suggests that platelets may also be involved in the recruitment of leukocytes, in particular monocytes and neutrophils, to the tumour microenvironment. Indeed, platelets contain a number of chemokines such as CCL3, CCL5, CXCL5 and CXCL8^72^ which as mentioned before have been demonstrated to recruit monocytes and neutrophils. Upon the formation of platelet-tumour cell aggregate, platelets were found to release CXCL5, resulting in CXCR2-dependent recruitment of neutrophils to the early metastatic niche in murine colon carcinoma and B16-F10 melanoma models.^73^ Similarly, these aggregates were also found to mediate monocyte recruitment into the metastatic niche in melanoma xenograft models.^74^ Interestingly, it has been recently demonstrated through intravital microscopy of the cremaster muscle that platelets adhered on the endothelium can also physically interact with circulating neutrophils and monocytes though CD40L/CD40 and P-selectin/PSGL-1 and guide them to their site of extravasation,^75^ thereby providing an additional potential mechanism for recruitment of neutrophils and monocytes to tumours. While still in its infancy stages, it is evident that platelets are involved in leukocyte recruitment to the tumour microenvironment and further investigations are required to determine their impact on melanoma disease progression and patient survival.

## Immunotherapy combination approaches for melanoma to optimise T-cell recruitment to tumours

With our increasing understanding of the mechanisms of immune cell entry into tumours and how the immune cell composition can affect treatment response, it is timely to start considering ways to selectively alter leukocyte recruitment to enhance treatment outcomes for melanoma patients. Indeed, a large number of clinical trials in which an immunotherapy backbone is combined with endothelium-modifying components are underway. These approaches will be discussed in more detail below. However, it is important to mention that although the effectiveness of immunotherapy may be improved under these conditions, increased numbers of adverse events are often reported. Therapeutic resistance even for combinations may also develop because tumour cells can potentially activate alternate pathways to evade the immune attack.^76^

### Anti-angiogenic therapy

The initial concept of anti-angiogenic therapy, in particular anti-VEGF/VEGFR therapy, was to achieve tumour growth inhibition by blocking blood vessel development in tumours and thereby depriving tumour cells of oxygen and nutrients.^77^ Despite success in preclinical animal models, anti-angiogenic monotherapy has failed to demonstrate an improvement in overall survival in melanoma clinical trials.^77^ Nonetheless, there has recently been a conceptual shift to begin using these agents to restore the tumour vasculature to its normal state, a process known as vascular normalisation. Indeed, VEGF-signalling inhibition with the small-molecule VEGFR inhibitor sunitinib in B16 murine melanoma models has been found to promote vascular normalisation, restore adhesion molecule expression and enhance CXCL10 and CXCL11 expression, which together increased CD8^+^ T-cell infiltration.^78^ Although these anti-angiogenic agents show limited therapeutic efficacy as monotherapies, their efficacy may be improved by using them in combination with immunotherapy. In a phase 1 clinical trial combining ipilimumab and the VEGF inhibitor bevacizumab (NCT00790010), the ECs of metastatic melanoma patients exhibited enhanced ICAM-1 and VCAM-1 expression post-treatment compared to pre-treatment, resulting in enhanced CD8^+^ T-cell infiltration.^79^ Another multi-targeted VEGFR kinase inhibitor, lenvatinib, which is Food and Drug Administration approved for thyroid cancer but has only had modest activity in melanoma, is currently in combination therapy trial with the anti-PD-1 drug pembrolizumab (NCT02501096).

### Low-dose radiation and chemotherapy

Before the development of targeted therapies and immunotherapy for melanoma, chemotherapy and radiation had been used as first-line and palliative therapies but with modest efficacy and no demonstrated improvements in overall survival. Of interest, chemotherapy and low-dose radiation have both been shown to promote changes to the tumour endothelium, suggesting that they may have synergistic activity with immunotherapy. In particular, chemotherapy with dacarbazine increased intratumoral CCL5, CXCL9 and CXCL10 expression, which was associated with increased tumour control in melanoma patients,^55^ while low-dose radiation promoted vascular normalisation and enhanced CXCL9, CXCL10 expression, together allowing for enhanced T-cell entry *in vivo*.^80^ In addition, low-dose radiation was also found to polarise M2 TAMs to an M1 phenotype, which through the reduction of VEGF production was also found to promote T-cell infiltration.^81^ Clinical trials revealed that combination therapy with ipilimumab and the chemotherapy agent fotemusine demonstrated long-term effects.^82^ Although phase 1 clinical trials of radiation and ipilimumab in metastatic melanoma patients demonstrated that only 18% of patients had partial response as best response, the therapeutic effects were enhanced by combining radiation, anti-CTLA-4 and anti-PDL-1 in murine melanoma and other tumour models.^76^ There are many ongoing Phase II clinical trials investigating combinations of ipilimumab (NCT01565837, NCT02107755) with hypofractionated high-dose stereotactic radiotherapy in metastatic melanoma patients.

### BRAFi

In addition to their direct tumoricidal activity, the BRAFi inhibitors, vemurafenib and dabrafenib, have also been found to promote changes in leukocyte recruitment. Metastatic melanoma patients receiving the BRAFi, vemurafenib, demonstrated increased CD8^+^ T-cell infiltration.^83^ The underlying mechanism was addressed *in vivo*, where BRAFi suppressed melanoma VEGF production and enhanced the infiltration of adoptively transferred T-cells in a murine melanoma model.^84^ This highlights the potential of combining immunotherapy with BRAFi as a means of increasing CD8^+^ T-cell infiltration. Phase II trials revealed that sequencing administration of vemurafenib followed by ipilimumab^85^ had manageable safety, and patients treated with vemurafenib for 6 weeks remained responsive to ipilimumab treatment and vemurafenib retreatment. *In vivo*, anti-PD-1 and BRAFi combination therapy demonstrated synergistic effects in a BRAF^V600E^/Pten^−/−^ murine model, with significantly increased CD8^+^ T-cell infiltration and survival compared to BRAFi treatment alone.^86^ In terms of combining BRAFi and CAR T-cell therapy, dabrafenib has not been found to affect GD2-specific CAR T-cell function *in vitro*.^87^

## Preclinical approaches to optimising the intratumoral leukocyte profile of melanoma

In addition to the aforementioned clinical studies, many other approaches are currently being developed in animal models, which aim to improve the efficacy of immunotherapy by rationally combining with ways of modulating T-cell recruitment to tumours. While these are yet to be translated into clinical trials, they are discussed here to highlight the exciting future possibilities for clinical immunotherapy.

As discussed above, the tumour endothelium expresses several molecules that inhibit CD8^+^ T-cell infiltration, and therefore can be targets for inhibition to enhance CD8^+^ T-cell entry. *In vivo*, CD73 blockade and anti-CTLA-4 co-therapy enhanced CD8^+^ T-cell infiltration in a B16-F10 melanoma model and inhibited tumour growth.^88^ Similarly, in multiple *in vivo* solid tumour models, combining ACT with CD73 blockade, FasL blockade or endothelin B receptor inhibition led to enhanced T-cell homing and infiltration into tumours, resulting in tumour regression.^36, 42^

Another approach to enhance tumour T-cell infiltration is by increasing the expression of CCR5 and CXCR3 ligands within the tumour microenvironment. Tang and colleagues proposed the use of an antibody-guided LIGHT fusion protein to enhance the efficacy of anti-PD-1 therapy.^89^ By specifically targeting LIGHT to tumour tissues to stimulate lymphotoxin β receptor (LTβR), the expression of CCL3, CCL4, CCL5, CXCL9 and CXCL10 was increased in tumour cells, resulting in enhanced T-cell infiltration and responsiveness to PDL-1 checkpoint blockade treatment.

Improvements in our understanding of the tumour microenvironment could be used for genetic engineering enhancements of CAR T-cells that might facilitate their entry into tumours. In particular, T-cells could be engineered to express receptors to the aforementioned factors, which are upregulated with disease progression and which also recruit neutrophils and monocytes. Indeed, when T-cells were engineered to express CXCR2, their recruitment to B16 murine melanoma overexpressing CXCL1 was increased, resulting in greater tumour regression compared to T-cells lacking CXCR2 overexpression.^90^

Apart from enhancing CD8^+^ T-cell infiltration, another approach is to inhibit TAM or TAN infiltration. Reducing TAM recruitment either via depletion of TAM or inhibition of CCL2 has been found to decrease tumour burden in a human melanoma xenograft model.^64^ Similarly, when neutrophil infiltration was inhibited with CXCR2 inhibition, tumour regression was observed in a lung tumour xenograft model.^91^

The observed plasticity of TAM and TAN polarisation from pro-tumorigenic M2/N2 phenotypes to anti-tumorigenic M1/N1 phenotypes could be exploited for therapeutic purposes. It has been demonstrated in murine models of lung cancer that combining TAM activation using the macrophage activation agent DMXAA with immunotherapy increased both M1/M2 TAM ratios and CD8^+^ T-cell infiltration, resulting in regression of established tumours.^92^ Alternatively, combination therapy involving transforming growth factor beta blockade and immunotherapy in murine models of mesothelioma and lung cancer increased expression of CXCL1, CXCL2, CXCL5, GM-CSF and ICAM-1 in tumours, leading to accumulation of N1 TAN, as well as increased levels of CCL5, CXCL9, CXCL10, leading to enhanced CD8^+^ T-cell infiltration, which together resulted in tumour regression.^19, 93^

It is important to note that success in animal models does not always translate to clinical studies because of discrepancies between *in vivo* models and human studies. For example, although mice were found to be responsive to the macrophage activation agent DMXAA (5,6-Dimethylxanthenone-4-acetic acid; Vadimezan), its activity in human leukocytes was less pronounced, resulting in unsuccessful clinical trials.^92^ Interestingly, it was eventually found that the receptor for DMXAA is the STING receptor, which is different in humans,^92^ demonstrating how pre-clinical studies could lead to the understanding of mechanisms later implied in humans. In addition, *in vivo* models are often simplified and performed under tightly controlled conditions, which does not always recapitulate clinical conditions complicated by such factors as genetic variation, age differences and co-morbidities.

## Vasculogenic mimicry channels: an alternative route of leukocyte entry?

Although this review has so far focussed on the conventional tumour blood vessels formed by ECs, it is worth noting that melanoma cells themselves have also been reported to mimic EC behaviour. Most notably, melanoma cells can align along a deposited basement membrane forming interconnecting vessel-like structures with lumen, a process known as vasculogenic mimicry (VM). First described in 1999 in uveal melanoma,^94^ VM is considered a signature of aggressive cancers, and the occurrence of VM has now been found to correlate with poor prognosis in many cancers such as cutaneous melanoma, breast cancer and glioblastoma.^95^ Moreover, it is observed that VEGF inhibition resulted in adaptive resistance in melanoma xenografts, in turn leading to increased VM.^96^ This finding could help explain why anti-angiogenic monotherapies for melanoma have not been clinically successful, and indicates the biological significance of these VM vessels.

Interestingly, melanoma cells have also been reported to express some of the adhesion molecules and chemokines that are expressed by ECs to recruit leukocytes,^97, 98, 99^ some of which also appear to be expressed by VM-competent cells.^100^ The expression of these molecules is primarily thought to allow circulating tumour cells to interact with blood vessels and enter tissues to establish new metastases. However, an intriguing additional possibility is that these molecules allow melanoma cells lining VM channels to actively recruit specific immune cell populations from the circulation. This is an area of active investigation within our laboratory, and indeed, preliminary studies have revealed that leukocytes can roll, adhere and transmigrate across monolayers of VM-competent melanoma cells. This reveals a previously unknown mechanism for melanomas to directly control the immune composition of the tumour microenvironment.

Our rapidly growing knowledge of VM suggests several novel approaches to melanoma therapy. One obvious tactic is to block the formation of VM channels directly and thereby reduce the tumour's access to a blood supply, for example using the VM-targeting small-molecule inhibitor CVM-1118, currently under clinical development for several malignancies.^94^ On the other hand, pre-existing VM vessels may be manipulated to become efficient routes of entry for CD8^+^ T-cells, such as CAR T-cells or the activated T-cells induced by checkpoint blockade immunotherapy. However, there is much still unknown in this area and substantial further studies are still required.

## Conclusion

With a large body of pre-clinical research, and a rapidly increasing body of data collected from clinical trials with immunotherapeutic agents, we are now gaining a better understanding of how these treatments affect the melanoma microenvironment, and the factors which affect responsiveness to treatment are beginning to be revealed.

However, there are still several significant gaps in our knowledge, especially regarding the recruitment of TAM and TAN, where virtually all of our current knowledge is derived from animal models and these results are yet to be supported with clinical studies. Given the increasing evidence for the importance of TAM and TAN in mediating melanoma progression and therapy response, there is great value in further deciphering and understanding these processes.

Although we have chosen to focus this review on melanoma, many of the mechanisms and paradigms discussed will also hold true with other cancer types. Indeed, some of the checkpoint inhibitors have been recently approved for the treatment of renal cell carcinoma and non-small cell lung cancer, with hundreds of clinical trials currently open to test these treatments as single agents or in combination therapies for other cancer types. Unfortunately, as for melanoma, it is often observed that only a subset of such patients respond. Combining immune checkpoint blockade with ways of improving CD8^+^ T-cell recruitment to tumours may increase tumour response rates. In addition, although CAR T-cell therapy has shown great promise in the treatment of blood cancers, the extension of this approach to solid tumours is still very much at the experimental stage.^6^ Rational approaches to ensure that CAR T-cells effectively enter the tumour microenvironment may help to change this.

## Figures and Tables

**Table 1:** EC adhesion molecules implicated in the leukocyte recruitment cascade and their respective receptors/ligands on leukocytes

*Step of leukocyte recruitment cascade*	*Adhesion molecule on endothelium*	*Receptor/ligand on leukocytes*	*Receptor/ligand expression status on leukocyte subsets*			
			*Naive T cells*	*Memory T cells*	*Monocytes*	*Neutrophils*
Rolling	P-selectin	PSGL-1	✓	✓	✓	✓
	E-selectin	PSGL-1	✓	✓	✓	✓
		ESL-1	×	✓	✓	✓
	MAdCAM-1,	L-selectin	✓	×	✓	✓
	PNAd					
Firm adhesion	ICAM-1, ICAM-2	LFA-1 (CD11a/CD18; αLβ2 integrin)	✓	✓	✓	✓
		Mac-1 (CD11b/CD18; αMβ2 integrin)	×	×	✓	✓
	VCAM-1	VLA-4 (α_4_β_1_ integrin)	✓	✓	✓	×
		α4β7 integrin	×	✓	×	×
	MAdCAM-1	α4β7 integrin	×	✓	×	×
Transmigration	PECAM-1 (CD31)	PECAM-1	✓	×	✓	✓
	JAM-A	LFA-1 (CD11a/CD18; αLβ2 integrin)	✓	✓	✓	✓
	JAM-B	VLA-4 (α_4_β_1_ integrin)	✓	✓	✓	×
	JAM-C	Mac-1 (CD11b/CD18; αMβ2 integrin)	×	×	✓	✓

Abbreviations: ESL-1, E-selectin ligand-1; ICAM, intercellular adhesion molecule; JAM, junctional adhesion molecule; LFA-1, leukocyte function-associated antigen-1; MAdCAM-1, mucosal vascular addressin cell adhesion molecule-1; PECAM-1, platelet/endothelial cell adhesion molecule-1; PNAd, peripheral node addressin; PSGL-1, P-selectin glycoprotein ligand-1; VCAM, vascular cell adhesion molecule; VLA-4, very late antigen-4.

Adapted from refs 29,37.

**Table 2:** Chemokines currently demonstrated to recruit CD8+ T-cells, TAM and TAN to melanoma, and their respective receptors on leukocytes. This summary is based on a combination of human clinical studies and murine models^53,57–60,63,64,52^

*Leukocyte population*	*Chemokine receptors*	*Cognate chemokines*	*Source of Data*
CD8+ T-cells	CCR5	CCL3, CCL4, CCL5	Human clinical studies and murine models
	CXCR3	CXCL9, CXCL10, CXCL11	
TAM	CCR2	CCL2	Murine models
TAN	CXCR1, CXCR2	CXCL1, CXCL2, CXCL6, CXCL8	Murine models

Abbreviations: TAM, tumour-associated macrophages; TAN, tumour-associated neutrophils.
